# The molecular mechanisms of increased radiosensitivity of HPV-positive oropharyngeal squamous cell carcinoma (OPSCC): an extensive review

**DOI:** 10.1186/s40463-018-0302-y

**Published:** 2018-09-21

**Authors:** Changxing Liu, Daljit Mann, Uttam K. Sinha, Niels C. Kokot

**Affiliations:** 1 USC Tina and Rick Caruso Department of Otolaryngology-Head and Neck Surgery, Keck Medicine of USC, University of Southern California, Los Angeles, CA 90033 USA; 2 Keck School of Medicine, University of Southern California, Los Angeles, CA 90033 USA

**Keywords:** HPV-positive oropharyngeal squamous cell carcinoma, Radiosensitivity, HPV, P53

## Abstract

Head and neck carcinomas (HNCs) collectively are the sixth most common cancer with an annual incidence of about 400,000 cases in the US. The most well-established risk factors for HNCs are tobacco and alcohol abuse. With the increasing public awareness, the incidence of HNCs is decreasing. But there is an increasing incidence of oropharyngeal squamous cell carcinoma (OPSCC) has been observed during the last decade. This phenomena is associated with persistent infection with high-risk HPV. HPV associated OPSCC patients tend to be younger males of high socioeconomic status. The increasing incidence causes a significant loss to social resources, given that it’s reported that HPV associated OPSCC represents about 60% of OPSCC cases. There is a growing amount of data supporting the hypothesis that HPV-associated OPSCC has a better survival rate due to a higher sensitivity to chemotherapy and radiotherapy as compared to HPV-unrelated OPSCC. Although the HPV positivity is associated with increased radio-sensitivity, the underlying mechanisms are not yet fully understood. This review summarizes the current knowledge on the effects of HPV infection and its carcinogenesis on the radiosensitivity of OPSCC, from the molecular to histologic level, providing a comprehensive insight of this special tumor entity.

## Background

Head and neck carcinomas (HNCs) collectively are the sixth most common cancer with an annual incidence of ~ 400,000 cases [1], and represent about 3.5% of all malignant tumors in western societies [2, 3] and other parts of the world. Nearly 90% of these cancers are head and neck squamous cell carcinomas (HNSCCs). The most well-established risk factors for HNSCC are tobacco and alcohol abuse [4]. HPV involvement in head and neck carcinogenesis was initially reported 30 years ago [5]; however, it was just recently recognized as an emerging risk factor for oropharyngeal squamous cell carcinoma (OPSCC) [6].

OPSCC arises in the oropharynx, the middle region of the throat that includes the soft palate, the base of the tongue, the tonsils, and the lateral and posterior walls of the throat. According to the American Cancer Society’s most recent estimates for 2016, in the United States, about 48,330 people are estimated to get oral cavity or oropharyngeal cancers, and an estimated 9570 people will die of these cancers. An increasing incidence of OPSCC has been observed during the last decade [7–10]. This rise in incidence is mostly occurring in individuals aged 40–55 years, without environmental risk factors, and is associated with persistent infection with high-risk HPV [11]. HPV-positive OPSCC patients tend to be younger than HPV(−) patients [12]. Tonsil and oropharyngeal cancers increased in male predominance over the last 30 years, despite a decline in smoking, which may be linked to the decreasing proportion of HPV-negative cancers; while changes in sexual activity may be reflected in increasing proportion of HPV-positive cancers [11]. Recently, HPV associated OPSCC represents about 60% of OPSCC cases compared to 40% in the previous decade.

There is a growing amount of data supporting the hypothesis that HPV-related tumors have a better survival rate due to a higher sensitivity to chemotherapy and radiotherapy as compared to HPV-unrelated HNSCC [12]. This significant difference in etiology and outcomes has been recognized by the AJCC, and new staging guidelines have been released to reflect the data [13]. DNA damage in HPV-related and HPV-unrelated HNSCC cell lines occurs by different mechanisms, which illustrate the reasons for the increased sensitivity of HPV-related OPSCC [14]. HPV positivity is associated with increased radio-sensitivity in probably multiple, not yet understood pathways [15–20]. This review summarizes the current literature on the effects of radiation therapy on OPSCC cell lines and how HPV infection alters these mechanisms to create a higher sensitivity to treatment.

## Radiation in OPSCC treatment

Radiation has a significant role in the management of head and neck squamous cell carcinomas (HNSCCs). Strategies to use radiation for the treatment of HNSCC have improved significantly during the past two decades, primarily through the use of IMRT techniques and through the addition of chemoradiotherapy. Radiation delivered postoperatively in the adjuvant setting significantly improves eradication of locoregional disease, and organ-sparing approaches for pharyngeal and laryngeal cancers that rely on chemoradiation as the primary treatment modality yields high rates of disease control [21]. The goal of radiation as a cancer treatment is to preferentially kill cancer cells over normal cells. This is achieved by both direct and indirect damage at different cellular levels of radiated tissue. Radiotherapy can injure nuclear DNA, nuclear membrane, organelles, and the cell membrane.

Nuclear DNA is the major target of radiation, and the two principal mechanisms of radiation induced damage are direct ionization damage to DNA, and indirect damage by free radicals formed in the microenvironment of the DNA by water radiolysis. The indirect damage from free radicals plays a larger role in inducing DNA damage, so it is important to recognize that oxygen is required to trigger the injury. This is one of the reasons that intra-tumor hypoxia can induce radioresistance [22].

Recent findings from the Radiation Therapy Oncology Group (RTOG) 0129 trial demonstrated that patients with oropharyngeal squamous cell carcinoma (OPSCC) that is positive for human papillomavirus (HPV) had significantly improved overall survival and progression-free survival, whereas HPV-negative OPSCCs treated with primary radiation therapy and cisplatin had significantly reduced locoregional control and overall survival [15]. These findings have opened the window for personalized medicine based on the molecular signature of the tumor. In fact, 2 RTOG trials were conceived based on this concept, and those trials were designed to evaluate the role of treatment de-intensification in HPV-positive OPSCCs (RTOG 1016) and the role of treatment intensification in HPV-negative OPSCCs (RTOG 1221). Thus, HPV positivity or expression of the p16 protein, a surrogate marker for HPV infection in OPSCC, appears to be a prognostic biomarker and is a potential predictive biomarker in HNSCC.

## Radiobiology of cancer treatment

**Fig. 1:**
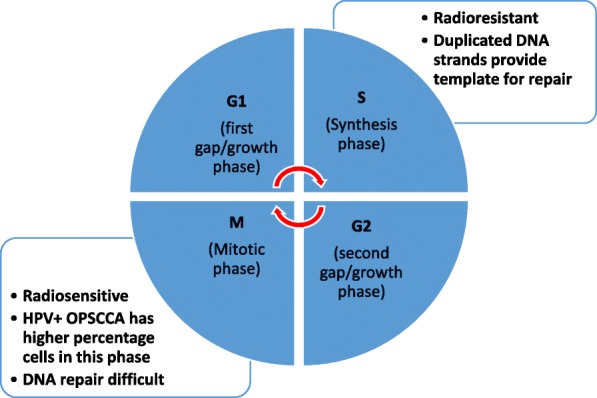
Cells have different radiosensitivities during different cell cycle phases. Cells during M phase have the highest sensitivity, as all chromosomes are condensed and packed in the middle of nucleus to form a bigger target, all the DNA repair mechanisms are shut down. On the contrary, all the chromosomes are very loose, and the DNA repair machineries are activated during S phase, so cells during S phase are most resistant to radiation. HPV+ OPSCC pass G1 and S phases quickly and accumulate at M Phase

Cancer cells have different radiosensitivities during the various cell cycle phases. For instance, during the M phase, all chromosomes are condensed and located in the middle of nucleus to form a larger target. All the DNA repair mechanisms are shut down, cells can lose large fragments of DNA, and any injury will be fixed in place. This contributes to cells having the highest sensitivity to radiation during the M-phase of the cell cycle. In contrast, during the S phase, DNA repair mechanisms are activated, and chromosomes are loosely arranged in the nucleus allowing cells in the S phase to be the most resistant to radiation [23] (Fig. 1).

There are several classes of DNA damage that can occur with radiotherapy, which include base pair injury, base pair deletion, cross linkage, single strand break or double strand break. When DNA is damaged by radiation, the first response for the cell is to arrest the cell cycle, then check and repair DNA injuries [22]. The result may lead to either repair of the insult or some type of cell death. Minimal damage can be repaired and the cancer cell will resume proliferation and duplication. Cancer cells have several different mechanisms to repair the damage caused by radiation, and the mechanism that is used depends on the class of induced damage. However, if the damage is severe and unable to be repaired, apoptosis or other cell death pathways will be initiated. Double strand DNA breaks most commonly lead to cell death as all other types of injury are more likely to be repaired [22].

DNA double strand breaks (DSBs) are repaired primarily by two mechanisms: non-homologous end-joining (NHEJ) and homologous recombination (HR) [24]. The mechanism of repair is dependent on the type and time of injury. NHEJ is utilized for simple and primary DSBs, and HR for clustered and secondary DSBs that occur post-radiation. The cell cycle phase at the time of injury is an important factor as well. For example, damage induced during G1 phase tend to be repaired with NHEJ, and during the S and G2 cell cycle phases are more likely to be repaired by HR. If the DNA damage is unable to be repaired, the cell pathways will induce cell apoptosis, senescence, or mitotic catastrophe. The final cell pathway depends on the functional status of cell cycle checkpoints and p53 [25].

## Role of p53 and Rb protein in cell cycle regulation

Normal cells have 3 checkpoints during a cell cycle: G1, G2 and M (spindle assembly) checkpoints. These checkpoints confirm that cells are ready to continue proliferation without error. G1 checkpoint controls the passage of G1 into S phase. It mainly verifies that the size of the cell and the environment are correct and favorable to continue. The G2 &M checkpoints mainly prevent the cell from entering mitosis (M phase) if the genome is damaged, which is almost exclusively internally controlled [26].

P53 is a transcription factor known to be an important regulator in DNA damage and repair. Phosphorylation of p53 permits downstream interaction with additional transcriptional cofactors, and is ultimately important for activation of target genes responsible for cell cycle arrest, DNA repair, apoptosis and senescence [27]. P53 is a substrate for both the ATM/ATR kinases, as well as for CHK1/CHK2 [28]. Both ATM and ATR belong to a structurally unique family of serine-threonine kinases. P53 induction by acute DNA damage begins when DNA double-strand breaks trigger activation of ATM or ATR kinases [29]. Phosphorylation of p53 occurs at several sites. As a consequence, activation enhances p53 stability and activity, and regulates the transcriptional activation of a number of downstream target genes, such as p21, GADD45, 14–3-3, Bax, and Mdm2 [29]. This process acts as a major regulator in cell cycle arrest, apoptosis, senescence, DNA repair, metabolism, and even invasion and metastasis. This role has led to p53 to also be known as the molecular node in the radiation response [28].

Another important protein family in cell cycle regulation is the Rb protein family, which plays a pivotal role in negative control of the cell cycle by acting as a major G1 checkpoint controller, blocking S-phase entry and cell growth. The Rb protein family includes three members which repress gene transcription required for transition from G1 to S phase, by directly binding to the transactivation domain of transcription factor E2F. When cells are activated, Rb protein will be phosphorylated and releases E2F. In turn, E2F-dependent genes will be transcribed and accelerate cell passage from G1 checkpoint into S phase. When DNA is damaged from radiation therapy, p53 will be phosphorylated and stabilized by ATM or ATR. It will then inhibit Rb protein from releasing E2F via p21 protein. This results in arrest of cell cycle at the G1 checkpoint [30].

## P53 induced apoptosis/senescence

When the damage is too severe and the repair fails, the cell processes will be switched towards cell death. P53 can induce cell apoptosis through an extrinsic or intrinsic pathway. However, radiation therapy primarily acts via the intrinsic pathway. In the intrinsic pathway, eventual accumulation of cytoplasmic P53 induces pro-apoptotic genes Bax, PUMA, and Noxa [31]. Bax will insert into the mitochondrial membrane, creating a permeable outer mitochondrial membrane. This leads to the release cytochrome c and triggers cell death through the caspase cascade. In the extrinsic apoptotic pathway, p53 will induce expression of apoptosis receptors CD95/Fas, KILLER/DR5, and the CS95/Fas Ligand. Activation of these receptors leads to downstream induction of the caspase cascade as well, ultimately leading to apoptosis and cell death [31].

Cells with greater expression of p53 are generally more prone to respond to radiation therapy and are considered to be more radiosensitive. Radiation induced apoptosis regulated by p53 is the main mechanism of cell death in cells of myeloid and lymphoid origin [27]. Radiation-induced intrinsic apoptosis occurs within a few hours following radiation exposure in the interphase before mitosis. However, if cells have already passed the interphase stage and are in M phase, other cell death mechanisms will be activated, including senescence and mitotic catastrophe [27].

**Fig. 2:**
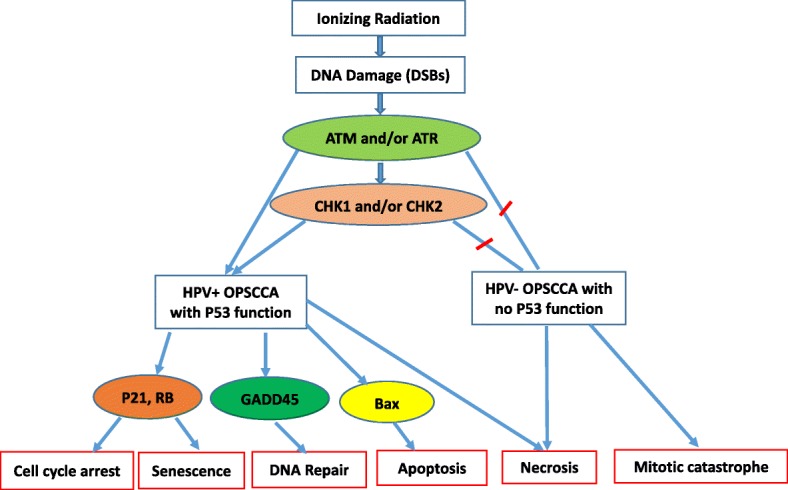
P53 is the most important regulator in DNA damage. P53 is a substrate for both the ATM and ATR kinases, as well as for CHK1 and CHK2. Phosphorylation of p53 allows its interaction with transcriptional cofactors, transcribes and activates its target genes for extensive cell responses, such as cell cycle arrest, DNA repair, apoptosis and senescence. When DNA damage is difficult to be repaired, it will induce cell apoptosis, senescence, or mitotic catastrophe. The final death pathway depends on the function of checkpoints and the status of p53

Senescence is a state of permanent cell cycle arrest which can be induced by radiation, and is another mechanism to prevent cancer cell growth. When DNA damage is irreparable, a chronic DNA damage response signal is produced to create cell cycle arrest. This state of arrest prevents damaged DNA from being propagated to daughter cells. Senescence caused by radiation is induced by p53 and p21. Senescent cells do not divide but may remain metabolically active. As a result, they may still possess the ability to secrete both tumor suppressing and tumor promoting factors which could lead to an inflammatory response to the tumor, or stimulate cancer cell growth [27, 28] (Fig. 2).

## Mitotic catastrophe

Mitotic catastrophe is an event in which cell death happens during mitosis or as a consequence of aberrant mitosis. There are two predominant proposed mechanisms for the induction of mitotic catastrophe. The first mechanism is from a combination of radiation induced DNA damage and dysfunction cell cycle checkpoints. Checkpoint inactivation generally occurs when tumor cells have mutated or inactivated p53. This would allow a cell with damaged DNA to progress passed the G2/M checkpoint. Since p53 is also important in DNA repair, mitotic catastrophe will be the more likely fate of tumor cells in this phase [32]. The second proposed mechanism is by hyperamplification of centrosomes. This will lead abnormal chromosome segregation and create cells with multiple nuclei, and or several micronuclei. Centrosome hyperamplification is also more prevalent in cells without functional p53, and results in mitotic catastrophe. These cells can survive for several days after initial radiation, but undergo cell death either by delayed necrosis, delayed apoptosis, or induced senescence. Mitotic catastrophe is considered to be the major cell death mechanism by which solid tumors respond to clinical radiotherapy [33].

## HPV in OPSCC radiotherapy

Human papillomavirus (HPV) is a small, non-enveloped DNA with a circular, double-stranded viral genome. Nearly all our knowledge of the mechanisms by which HPV causes cellular proliferation and neoplasia are from research of HPV in cervical cancer. This research can be extrapolated to HPV associated OPSCC, as the most common high-risk HPV genotypes in head in neck cancers is also HPV genotypes 16 and 18 [34]. Besides the obvious difference in anatomic location, there may be some difference in the natural history of HPV infection in cervical cells when compared to oropharyngeal cells, and in the manifestation of HPV associated neoplasia in the two tissues. From studies in women with cervical HPV infection, only 5–10 per 100,000 progresses to malignant cervical disease. On the other hand, preventative screening for oropharyngeal infection is not currently the standard of practice and therefore studies to compare cervical infection rates to head and neck infection rates are not available. However, the fundamental molecular mechanisms by which HPV encoded proteins circumvent cell-cycle control are likely to be the same, irrespective of tissue localization [35]. It is also important to understand that p16 overexpression is used as a surrogate marker to identify actively infected HPV cells, and has been incorporated into the new 8th edition AJCC staging guidelines [34, 13]. Immunohistochemistry assays are cost effective and widely available in most laboratories and therefore serves as an excellent surrogate marker [34]. However, more accurate testing for specific high risk genotypes should be considered to further classify responsiveness to therapy in future studies.

The infection and proliferation of HPV is unique. New HPV virions are only produced once the initially infected cell has undergone mitosis and one of the daughter cells has differentiated. HPV does not encode proteins directly responsible for replication of its own DNA. Instead, it uses the host cell replication machinery. HPV encoded proteins disrupt multiple cellular signaling pathways to maintain infected cells in a proliferative state that facilitates viral persistence and replication [34]. The primary viral proteins responsible for disruption of the host cell-cycle, and most significant in oncogenesis, are the E6 and E7 oncoproteins.

## E6/E7 Oncoproteins

Expression of both viral E6 and E7 oncogenes is consistently maintained in infected cells to inhibit the functions of p53 and Rb tumor suppressor pathways. E6 oncogene expression will lead to ubiquitination and subsequent degradation of p53 protein. This allows for dysregulation of both G1/S and G2/M cell cycle checkpoints leading to genomic instability. E6 oncoprotein also has the ability to activate cellular telomerase through transcriptional upregulation of the catalytic subunit of human telomerase. This allows the infected cell to maintain telomere length, which is an important step in cellular immortalization and transformation [36].

**Fig. 3:**
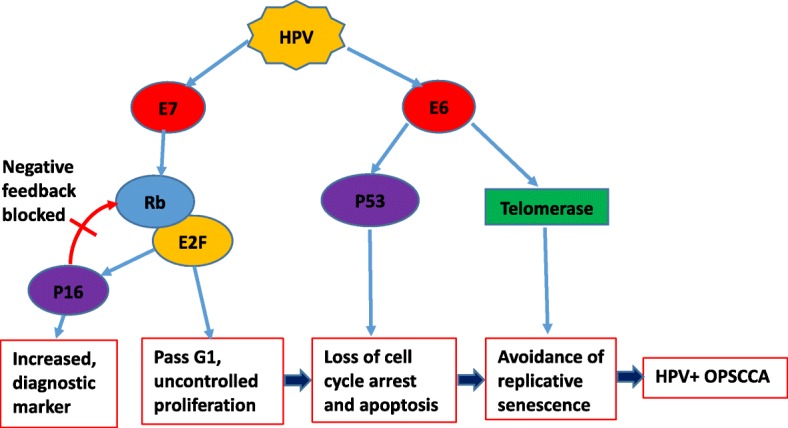
E6 and E7 are the main oncoproteins. They are encoded by HPV and disrupt or usurp multiple cellular signaling pathways to maintain infected cells in a proliferative state that facilitates viral persistence and replication. HPV E7 oncoprotein inactivates the Rb protein family, resulting in over-activation of E2F transcription factor with increased transition of cell from G1 to S phase and cell proliferation. Inactivation of Rb proteins also results in increased levels of p16, a marker for HPV infection diagnosis. E6 degrades p53 protein, deregulating cycle checkpoints, and activates telomerase through the transcriptional upregulation of an important subunit of human telomerase, maintaining the telomere length. E7 and E6 have complementary effects

HPV E7 oncoproteins have a complementary effect to E6 oncoproteins by binding and inactivating the Rb protein family. This results in overactivation of E2F transcription factor with upregulation of cell cycle genes, and transition of cell from G1 to S phase and an increase in DNA synthesis and cell proliferation. Inactivation of Rb proteins also results in increased levels of p16, an inhibitor of cdk4/cyclin D, by a feedback control mechanism. Therefore, high levels of p16 expression serves as a specific diagnostic biomarker for tumor infected with HPV [36] (Fig. 3).

## Pathologic and genetic profile differences

**Fig. 4:**
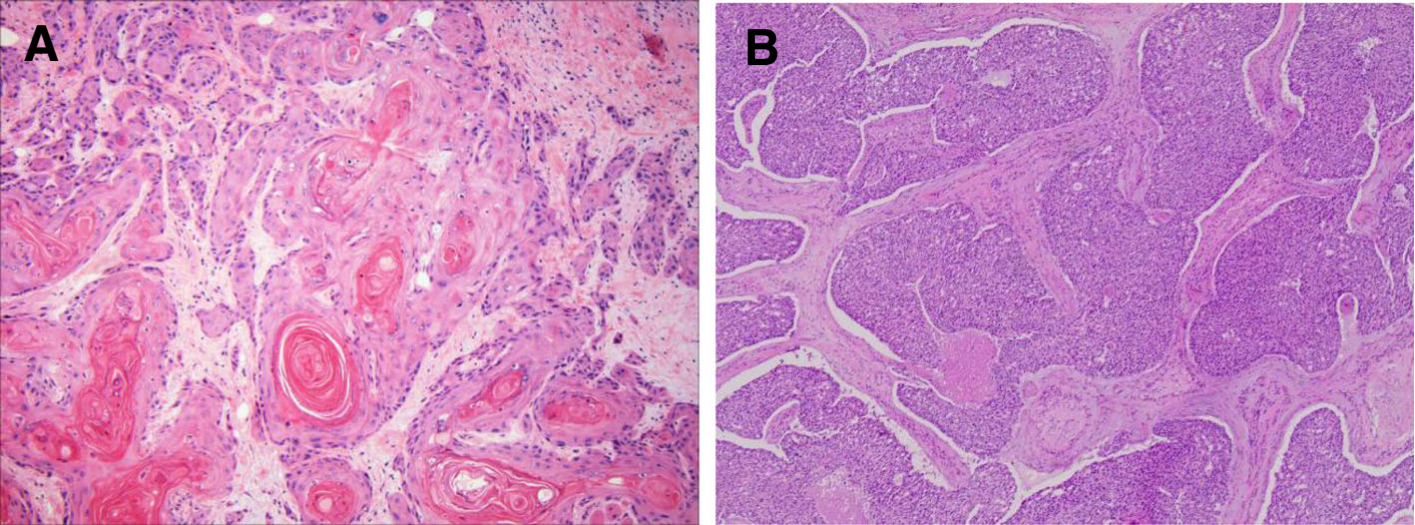
Histological characteristics of HPV-positive and HPV-negative OPSCC. HPV-positive OPSCC normally shows no surface dysplasia or keratinization, it has lobular growth with infiltrating lymphocytes, and often demonstrates baseloid variation. Photo (**a**) is HPV− OPSCC, keratinizing tumor cells with abundant pink cytoplasm in discrete nests. Photo (**b**) shows HPV+ OPSCC, nonkeratinizing hyperchromatic tumor cells with ill-defined borders, abundant mitoses and areas of necrosis

There are also some differences in pathological characteristics when comparing HPV-positive tumors to HPV-negative tumors. It has been shown that HPV-positive OPSCC normally shows no surface dysplasia or keratinization, has lobular growth with infiltrating lymphocytes, and often demonstrates baseloid variation. On the other hand, HPV negative OPSCC, histologically shows keratinizing tumor cells with abundant pink cytoplasm composed in discrete nests [35] (Fig. 4).

Gene profiling of HPV positive and HPV negative oropharyngeal cancer has shown that HPV-positive cancers have a significantly different genetic expression profile. Differences were particularly common among genes involved in DNA regulation and repair (CDKN2A, LIG1, MCM6, E2F, CDT1, PARP2, SMC4, CDC7, etc.), cell cycle (MLLT6, PTTG1, CUL4A, LIG1, SASS6, etc.), and chemotherapy/radiotherapy-sensitivity (CCND1, TYMS, STMN1, RBBP4) [37].

Epigenetic modifications mainly effect tumorigenesis by DNA methylation, histone modification and miRNA expression. These mechanisms and their role in OPSCC is thoroughly summarized in a recent review by Lindsay et al. [38]. Briefly, HPV- positive OPSCC has been shown to have increased areas of DNA methylation when compared to HPV-negative tumors which are associated with genome wide hypomethylation. One proposed mechanism of this difference is from DNA methyltransferase (DNMT) dysregulation as there is increased expression of the protein in HPV positive tumors, likely from the viral oncoproteins E6 and E7 discussed above. The authors also discuss the histone methyltransferase EZH2 as a possible HPV-positive tumor marker that may have a role in regulating cell proliferation. EZH2 is another downstream product of the E7 oncoprotein and is therefore closely associated with HPV positive malignancies. There is still further research required on the topic of micro and non-coding RNA and its epigenetic effects on OPSCC [38]. There are a few chemotherapy agents under study that target these epigenetic differences, however there are no trials summarized for OPSCC specifically and they show poor activity against solid tumors. Although there may be epigenetic difference between HPV- positive and HPV- negative OPSCC, further research is required to determine outcomes of targeted radiation therapy and if the differing response can be partially attributed to the epigenetic profiles.

## HPV-positive OPSCC response to radiation

HPV-positive cancer cells have different gene profiles. Studies show that there are no significant differences in the average number of mutated genes, however, the gene expression levels are significantly different (347 differentially expressed genes) in HPV-positive vs HPV-negative cancers [39]. Differences were particularly common among genes involved in DNA regulation and repair, cell cycle regulation, and chemotherapy/radiotherapy-sensitivity. These differences allow for increased sensitivity to radiation in HPV-positive cells [39]. This leads us to the main mechanisms that lead to an improved response to radiation therapy: Altered DNA repair, reduced hypoxic regions, and increased cellular immune response.

In HPV-positive oropharyngeal cancer cells, p53 is still wild type and has normal functions. Although the concentration is low due to degeneration induced by E6 oncoprotein, it remains detectable and can be induced with radiation. When radiation breaks down double stranded DNA, p53 will be activated as discussed above. In HPV-negative OPSCC, p53 is mutated and cell cycle arrest will not occur. However, in HPV-positive tumors, there remains a basal level of functioning p53. This will activate cell cycle arrest and induce quick cell apoptosis if the DSBs are severe [40]. Increased p53 levels should also allow for a better response to radiation therapy in HPV-positive cells [41]. However, in one study which looked at radiation effects on 5 cell lines of HPV positive tumors, there was minimal evidence of apoptosis at the dosage of radiation studied, and instead found a substantial number of cells arrested in G2 phase associated with DNA DSB [42]. The authors concluded that the increased sensitivity was therefore more likely to be from the inability to repair DSB caused by the radiation. Future studies will need to address these differing opinions when a greater number of cell lines are available to study.

As previously discussed, P16 is over-expressed in HPV-positive OPSCC, which will impair DNA repair system by precluding Rad51 from access to the DNA damage site [43]. This causes a shift in the repair mechanism from homologous to non-homologous, and will lead to more errors. Misrepair of damaged DNA may lead to increased cell apoptosis if functioning p53 is available.

Another reason for the increased sensitivity to radiation is the consistently activated E2F from HPV E7 oncoprotein degradation of Rb protein family. This will promote cell cycle advancement from G1 to S phase, and as a result, all HPV-positive cells will accumulate at G2/M phases. In this phase, cells are more sensitive to radiation as the chromosomes are more densely packed, and DNA repair function is weak [41, 42].

Oxygen is important for radiotherapy and the formation of free radicals. Most solid tumors have hypoxic centers, and cancer cells are resistant to radiation because oxygen is not available to from free radicals in the microenvironment of the DNA to cause damage. Also, oxygen is important in fixating injury induced by radiation. Under hypoxic conditions, DNA damage is reduced (mainly by −SH-containing compounds), which leads to cell survival. Hypoxia-inducible factor 1α (HIF1α) is stabilized under hypoxic conditions, which leads to the upregulation of genes involved in cell survival [44]. HPV-positive OPSCCs are less hypoxic than negative ones, and the lack of hypoxia inside HPV-positive tumors could contribute to the improved radiosensitivity [40, 45]. This theory is supported by a retrospective analysis of the DAHANCA 5 trials performed by Lassen et al., which concludes that hypoxic radioresistance may not be relevant in HPV associated tumors since there is no benefit from hypoxic radiosensitization [45].

HPV-positive tumors have stronger T cells tumor infiltration and the higher radiosensitivity of HPV-positive OPSCC might be partially due to a more effective immune response following radiation. Tumor cell injury and inflammation induced by radiation lead to the release of tumor antigens and HPV viral antigens, which can trigger a stronger immune response [33, 44, 46, 47]. These findings have been confirmed in studies of immune-incompetent mice with HPV positive tumors that show improved outcomes with transplant of wild type immune cells and improved outcomes in mice given an E6/E7 oncogene vaccination prior to treatment [48].

## Conclusions

Over the past few decades, the number of oropharyngeal cancers linked to HPV has risen dramatically. HPV DNA is now found in about 2 out of 3 oropharyngeal cancers and in a much smaller fraction of oral cavity cancers. HPV-positive OPSCC tends to respond favorably to radiotherapy when compared to HPV-negative OPSCC. DNA damage in HPV-related and HPV-unrelated HNSCC cell lines occurs by different mechanisms. Low level wild type p53 is the key molecule for the enhanced radiosensitivity of HPV-positive OPSCC. The enhanced responsiveness of HPV-positive OPSCC to radiotherapy may also be due to a higher cellular radiosensitivity secondary to cell cycle dysregulation and impaired DNA DSB repair, and other factors including immune response and fewer hypoxic regions. However, all pathways are still not completely understood and require further research.

## Abbreviations

DSB: Double strand breaks
HNCs: Head and neck cancers
HNSCC: Head and neck squamous cell carcinomas
HPV: Human papilloma virus
OPSCC: Oropharyngeal squamous cell carcinoma
